# AUV SLAM and Experiments Using a Mechanical Scanning Forward-Looking Sonar

**DOI:** 10.3390/s120709386

**Published:** 2012-07-09

**Authors:** Bo He, Yan Liang, Xiao Feng, Rui Nian, Tianhong Yan, Minghui Li, Shujing Zhang

**Affiliations:** 1 School of Information Science and Engineering, Ocean University of China, 238 Songling Road, Qingdao 266100, China; E-Mails: liangyan@126.com (Y.L.); fengxiao88702@sina.com (X.F.); nianrui_80@163.com (R.N.); sjzhang365@gmail.com (S.Z.;; 2 School of Mechanical & Electrical Engineering, China Jiliang University, 258 Xueyuan Street, Xiasha High-Edu Park, Hangzhou 310018, China; 3 Centre for Ultrasonic Engineering, Department of Electronic and Electrical Engineering, University of Strathclyde, 204 George Street, Glasgow G1 1XW, UK; E-Mail: minghui.li@ieee.org

**Keywords:** AUV, mechanical scanning imaging sonar, FastSLAM

## Abstract

Navigation technology is one of the most important challenges in the applications of autonomous underwater vehicles (AUVs) which navigate in the complex undersea environment. The ability of localizing a robot and accurately mapping its surroundings simultaneously, namely the simultaneous localization and mapping (SLAM) problem, is a key prerequisite of truly autonomous robots. In this paper, a modified-FastSLAM algorithm is proposed and used in the navigation for our *C-Ranger* research platform, an open-frame AUV. A mechanical scanning imaging sonar is chosen as the active sensor for the AUV. The modified-FastSLAM implements the update relying on the on-board sensors of *C-Ranger*. On the other hand, the algorithm employs the data association which combines the single particle maximum likelihood method with modified *negative evidence* method, and uses the rank-based resampling to overcome the particle depletion problem. In order to verify the feasibility of the proposed methods, both simulation experiments and sea trials for *C-Ranger* are conducted. The experimental results show the modified-FastSLAM employed for the navigation of the *C-Ranger* AUV is much more effective and accurate compared with the traditional methods.

## 1. Introduction

An AUV is an untethered underwater robot which can navigate itself in the complex undersea environment. They are widely applied in military missions, resource exploration and oceanographic surveys. Generally, a mobile robot estimates its own position and posture by testing the internal state or perceiving the external environment, and adjusts its position and realizes accurate positioning under the guidance of a prior map, but in unknown complicated underwater environments, the prior map is not always available, then the AUV can only perceive the environment through on-board sensors such as cameras and sonar, obtain the useful information to construct the environmental map and estimate the vehicle location [1,2]. The ability of simultaneously localizing a robot and mapping its surroundings accurately is a key prerequisite of truly autonomous navigation [3]. The SLAM algorithm was proposed by Smith, Self and Cheeseman [4]. It can be described as that a mobile robot is placed in an unknown environment and the robot incrementally builds a consistent map of this environment while simultaneously determining its location within this map [1]. In the past two decades, the SLAM problem has received significant attention, and currently there are several popular SLAM algorithms such as the extended Kalman filter (EKF), the sparse extended information filter (SEIF) and the Rao-Blackwellized particle filter (RBPF), *etc*.

Smith *et al.* [5] proposed an EKF-SLAM method based on the extended Kalman filter, which is simple and easy to operate and widely applied in engineering. Currently, it has become a relatively mature SLAM algorithm. The EKF-SLAM can be described as a posterior probability distribution parameterized by a state vector and covariance matrix. The algorithm mainly consists of two steps: prediction and update, which can be summarized as an iterative estimate and calibration process [6]. However, the EKF-SLAM suffers from two major problems: its computational complexity and data association. The covariance matrix in the EKF-SLAM contains the covariance between the robot and environment features [7], which need to be updated after each estimation and correction. As a result, it achieves *O*(*n*^2^) (the number of environmental features) computational complexity. To deal with the limitation, people divide the world into a handful of sub-maps; each contains *l* features [8–12]. Thus, computational complexity can be reduced to *O*(*l*^2^), but the trade-off is sacrificing the convergence speed.

Thrun *et al.* [13,14] proposed a SEIF method based on the inverse of the covariance matrix. This algorithm uses the information filter instead of the extended Kalman filter and at the same time adopts the extended Kalman filter to linearize the nonlinear motion and observation model [15]. The advantage of the SEIF algorithm lies in the small computational burden, which only takes up *o*(*kn*) storage space and of which the computational complexity in the process of global update is *o*(*k*^2^). Measurement and movement can be realized in a constant time. The weakness of the algorithm lies in the difficult data association, bad consistency and impracticable covariance [16].

Arulampalam *et al.* [17] proposed the particle filter (PF) algorithm. The particle filter is a kind of Monte Carlo method applying samples to present the probability density distribution, which can be employed to any state model, and when sample size tends to infinity, it can approximate any form of probability density distribution. Murphy *et al.* [18,19] introduced the Rao-Blackwellized particle filter as an effective means to solve the SLAM problem, which provides theoretical basis for the SLAM research using the particle filters more efficiently. Montemerlo *et al.* [20,21] put forward the FastSLAM based on particle filter algorithm, which was actually an improved instance of Rao-Blackwellized particle filter, and presented the nonlinear and non-Gaussian state distribution using particles for the first time. Compared with the EKF-SLAM, FastSLAM adopts each particle to represent a potential trajectory of the robot and a map of the environment, which partitions the SLAM posterior into a localization problem and an independent landmark position estimation problem. FastSLAM has some advantages, especially in data association. It can deal with multi-hypothesis association problems because of the independence of the particles. According to the work by Bailey *et al.* [22], the FastSLAM algorithm cannot guarantee the consistency of robot pose estimation. Increasing the number of particles can improve the consistency to a certain extent, but simultaneously it will increase the computational cost. Because the robot pose estimation relies on a particle filter, a significant aspect of the FastSLAM algorithm research is the selection of the particle sampling function and the preservation of particle diversity.

Currently, a lot of research groups have their own AUVs for studying SLAM algorithms. In the ACFR of University of Sydney, the *Oberon* AUV extracted the point features from the sonar image to construct the environmental map while it estimates the robot position using EKF-SLAM [23]. MIT also carried out SLAM tests utilizing sonar data [24]. They used the data obtained from the high resolution array (HRA) forward looking imaging sonar in the Naragansett Bay to extract the point features aiming at detecting the objects and its position on the seabed and locating the robot at the same time. Heriott Watt University developed a system composed of a Doppler log, three axis compass and side scan sonar to perceive the environment and implement the EKFSLAM algorithm when executing submarine exploration tasks [25]. Many works prove that SLAM algorithms can be employed as a substitute for absolute orientation means such as GPS or acoustic baseline system and can also cooperate with them to assist localization. In addition, most research so far has applied the EKF-SLAM algorithm.

Taking the advantages of the FastSLAM algorithm, a modified algorithm based on the improved particle filter (PF) is presented in this paper, which employed the data association method by combining the single particle maximum likelihood method with a modified *negative evidence* method, and rank-based resampling was also adopted to overcome the particle depletion problem. In addition, the velocity and heading angle were taken not only as the state variables, but also as the observation variables. The sea trial experiments are also carried out in Tuan Dao Bay (Qingdao, China) on our AUV platform, the *C-Ranger*. The AUV position was predicted through the information such as speed, heading angle that was collected by the on-board sensors, at the same time, the environment was perceived via the forward-looking sonar, and the appropriate environment features are extracted and used for the construction of an environmental global map. In particular, the main contribution of this paper lies in the development of a modified-FastSLAM algorithm, which can be used effectively to construct a point-feature map of the undersea environment and localize the AUV position simultaneously. This paper is organized as follows: Section 2 presents the framework of the *C-Ranger* AUV platform developed in our own laboratory as well as the on-board sensors carried by the vehicle. In Section 3 we present the modified-FastSLAM algorithm used in our AUV and simulation experiments. The sea trial results are given in Section 4 to verify the effectiveness of the algorithm. Finally in Section 5 and 6, discussion and conclusions of the proposed technique will be presented.

## 2. The C-Ranger AUV and On-Board Sensors

### 2.1. C-Ranger AUV

**Figure 1.**
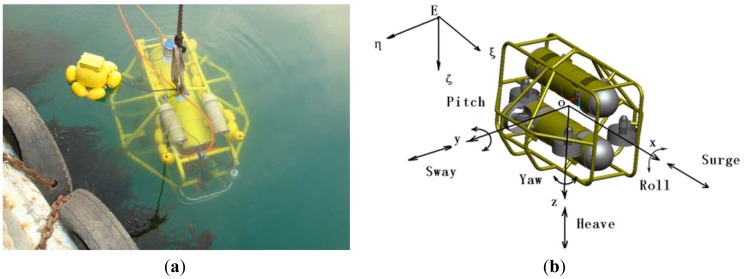
. (**a**) C-Ranger in deployment; (**b**) The coordinate system of the C-Ranger platform.

**Figure 2.**
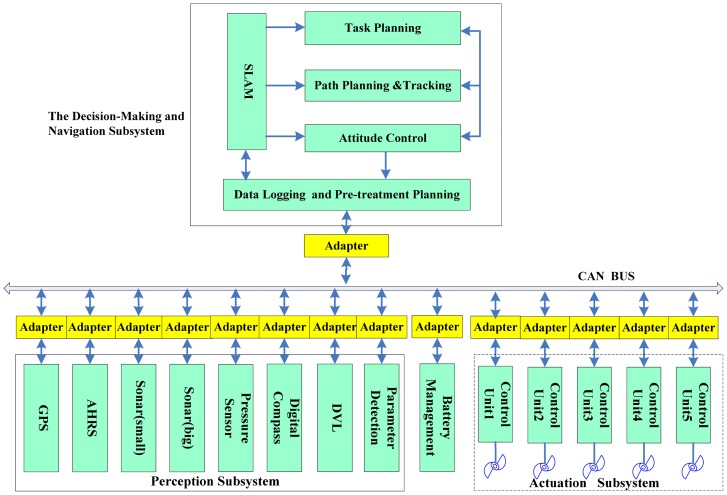
Control architecture of the C-Ranger.

The *C-Ranger* is an open-frame AUV with dimensions of 1.6 m × 1.3 m × 1.1 m (length, width and height), as shown in Figure 1. The control architecture of *C-Ranger* is illustrated in Figure 2.

The AUV has good maneuverability due to five DOFs, including surge, heave, roll, pitch, and yaw. The thrust system of this platform consists of five propeller thrusters, where two thrusters paralleling to the bow direction are installed in the abdomen to provide horizontal thrust, mainly for controlling the surge and yaw, while the other three thrusters, two of which are installed on both sides of the bow, and the remaining one is installed on the rear of the vehicle, are employed to provide vertical thrust to control the heave, roll, and pitch. The upper hull of *C-Ranger* is the instrument compartment, housing sensors, two industrial computers, communication module, internal monitoring module and other equipment, while the lower hull is the power and thrust system composed of lithium-ion batteries, power management module, motor-driver module, *etc*. The maximum speed of *C-Ranger* is 3 knots, and it can operate for up to 8 hours when fully charged (tested at a speed of 1 knot).

### 2.2. On-Board Sensors

A number of sensors are installed on the *C-Ranger*, and some of them are explicitly related to SLAM. These sensors are basically divided into two groups: the internal and the external. Internal sensors include digital compass, gyro, Attitude and Heading Reference System (AHRS) and pressure sensor. External sensors include mechanical scanning sonar, Doppler Velocity Log (DVL), altimeter, CCD camera and GPS.

**Figure 3.**
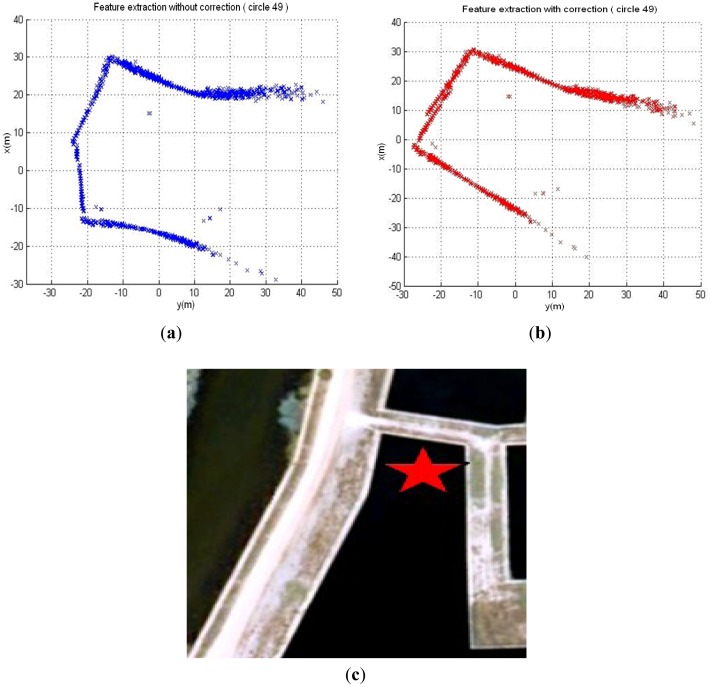
Effect of the vehicle motion on acoustic images. (**a**) Raw sonar image; (**b**) Corrected sonar image; (c) Zenithal view of the Abandoned Marina.

- Mapping-Related Sensor: Active Imaging Sensor
  A mechanically scanning forward-looking sonar (Super Seaking DST, Tritech) used for active sensing of environment features is installed at the front top of C-Ranger. It is the principal sensor of C-Ranger AUV. The operating frequency of the sonar is 675 kHz, and its working range is up to 300 meters.
  The transducer head of this kind of sonar usually needs a considerable period of time to perform a 360° rotation. This is an important issue that has to be taken into account when operating with such sonar mounted on an AUV, since the resulting acoustic images can get distorted as a consequence of the vehicle's motion. Generally, this effect can be ignored for low velocities but for higher velocities it has to be dealt with. Feature extraction is an important process, e.g., Forouher *et al.* [35] presented an interesting method to extract wall features for arbitrary shapes, which is different from that using position feedback to un-distort the data in our previous work [26]. The data in the 49th circle of the Abandoned Marina Dataset [27] is used to verify the effect of this correction method. Figure 3(a) shows the acoustic image built from the raw sonar data. Since the vehicle's motion has been ignored during the generation of the image, obvious distortion of the observed features appears when comparing it with the aerial image of the test scenario in Figure 3(c). The corrected image using the correction method is shown in Figure 3(b). Obviously the distortion of the image is almost canceled and a more accurate image is represented. The result demonstrates that the correction approach is effectual.
- Velocity Sensor: DVL
  The DVL (NavQuest600, LinkQuest) is used to provide the velocities of the vehicle relative to the seabed. In addition, the NavQuest600 can provide other information: pitch angle, roll angle, heading, altitude, depth, temperature and velocities relative to the ocean currents.
- Angular Sensors: AHRS and Gyro
  The AHRS (M2, Innalabs) is used to produce attitude information, and a gyro (VG951D) is used to measure angular velocity in the AUV navigation process. AHRS M2 is a low-cost high-performance inertial navigation system, and magnetic interference will not affect the accuracy of headings over short times.
- Positioning Sensor: GPS
  To evaluate the navigation performance of the *C-Ranger*, a high-precision and high-dynamic GPS receiver is employed. In the absence of SA, the positioning accuracy is up to 1.1 m (CEP), and the data update rate is up to 20 Hz. The GPS sensor can offer a benchmark to evaluate the estimation of robot trajectory.

## 3. The Modified-FastSLAM Algorithm

The goal of SLAM is to simultaneously localize a robot and determine an accurate map of the environment, that is, to determine the posterior *p*(**s^t^**,**Θ** | **z^t^**, **u^t^**, **n^t^**), where **s**^t^ = {*s_1_*,…,*s_t_*} is the path of the mobile robot and **Θ** = {*θ*_1_,…,*θ_m_*} is the map which contains *m* landmarks. The posterior is conditioned on all observations **z**^t^ = {*z*_1_,…,*z_t_*}, the sequence of control inputs **u**^t^ = {*u*_1_,…,*u_t_*} and data associations **n**^t^ = {*n*_1_,…,*n*_t_}. The data associations describe the mapping of observations to the landmarks in the map.

FastSLAM is an efficient algorithm for the SLAM problem which partitions the SLAM posterior into a localization problem and *m* independent landmarks estimation problems relying on the conditional independence. The posterior is given by the following factored format:

(1) $$p(s^{t},\Theta |z^{t},u^{t},n^{t})=p(s^{t}|z^{t},u^{t},n^{t})*\prod_{i=1}^{m}p(\theta_{i}|s^{t},z^{t},u^{t},n^{t})$$

In FastSLAM, a particle filter is used to approximate an ideal recursive Bayesian filter for the mobile vehicle's pose estimation *p* (**s^t^** ‖**z^t^**, **u^t^**, **n^t^**). The landmark locations posterior *p* (**Θ s^t^** ‖**z^t^**, **u^t^**, **n^t^**) are analytically calculated by EKF filters. Each particle in FastSLAM can be expressed as follows:

(2) $$S_{t}^{n}=\left\{s^{t,n},\mu_{t,1}^{n},\sum_{t,1}^{n}\cdots \mu_{t,N}^{n},\sum_{t,N}^{n}\right\}$$

In Equation (2)*n* indicates the index of the particle, **s**^t,n^ is the *n*-th particle's path estimate at time t, and $\mu_{t,i}^{n}$ and $\sum_{t,i}^{n}$ are the mean and covariance respectively of the Gaussian distribution representing the *i*-th landmark *θ*_i_, attached to the *n*-th particle. The FastSLAM algorithm has four basic steps that can be described as:

1. Sample a new pose for each particle.
2. Update the landmark observed with EKF in each particle.
3. Calculate an importance weight.
4. Importance resampling.

Our algorithm has made improvements to the FastSLAM2.0 and we call it modified-FastSLAM. This modified algorithm takes advantage of the FastSLAM 2.0 algorithm, which employs a data association method combining the single particle maximum likelihood method with a modified *negative evidence* method, and uses the rank-based resampling to overcome the particle depletion problem, besides taking the velocity and heading not only as the state variables, but also the observation variable.

### 3.1. Modified-FastSLAM Algorithm

- Sampling A New Pose for Each Particle
  The vehicle pose are sampled conditioned the motion model incorporating the most recent measurement data *z_t_* and control input *u_t_*, which can be denoted as:
  (3) $$s_{t}^{n}\sim p(s_{t}|s^{t-1,n},u^{t},z^{t},n^{t})$$
  The new particles are distributed according to:
  (4) $$p(s^{t-1}|u^{t-1},n^{t-1},z^{t-1})p(s_{t}|s^{t-1},u^{t},z^{t},n^{t})$$
  The distribution is referred to as the proposal distribution of particle filtering.
- Updating the Estimation of observed Landmark
  The FastSLAM represents the landmark estimates *p*(*θ* | **s^t^, z^t^, u^t^, n^t^**) in Equation (1) using EKFs. The posterior over the *i*-th landmark pose *θ_i_* can be acquired easily. Its computation depends on whether landmark *θ_i_* was observed at time *t*, that is, whether *i* = *n_t_*. The two kinds of circumstances are as follows. If the landmark is observed:
  (5) $$p\left(\theta_{i=n_{t}}|s^{t},z^{t},u^{t},n^{t}\right)\propto p\left(z_{t}|\theta_{n_{t}},s_{t},n_{t}\right)p\left(\theta_{n_{t}}|s^{t-1},z^{t-1},u^{t-1},n^{t-1}\right)$$
  The algorithm achieves the update Equation (5) using EKF.
  For *i* ≠ *n_t_*,the landmark posterior is unchanged:
  (6) $$p(\theta_{i\neq n_{t}}|s^{t},z^{t},u^{t},n^{t})\propto p(\theta_{i}|s^{t-1},z^{t-1},u^{t-1},n^{t-1})$$
  The classical solution to the data association problem is to choose *n_t_* such as it maximizes the likelihood of the measurement *z_t_* given all the data [21]. This procedure can be written as:
  (7) $${\hat{n}}_{t}=\underset{n_{t}}{\text{arg max}}p(z_{t}|s^{t},z^{t-1},u^{t},n_{t},{\hat{n}}^{t-1})$$
  Data association can be made on a per-particle basis because each particle represents a different predicted path. Montemerlo had put forward a per-particle ML (maximum likelihood) [28] data association method and adopted the “negative evidence” to remove spurious landmarks from the map. Each time a constant value is added to the log odds ratio if the landmark is observed and a constant value is subtracted from the log odds ratio if the landmark is not observed while it should have been. Once the ratio falls below a threshold, the landmark will be discarded. However, the accumulation of log odds ratio can't intuitively describe whether a landmark exists in the map or not. The setting of the threshold does not have a reliable standard, in addition, the algorithm does not set an acceptance gate for the new observations, which causes all new observed landmarks to join the map, and some false landmarks will influence the accuracy of localization and the map estimate [29].
  In order to correctly identify the false landmarks, and ensure accurate data association, this paper sets information variables to track observed landmarks and judges the authenticity of landmarks in the map in accordance with an acceptance gate and discard gate.
  All the observed landmarks are added into a preprocessing landmark set and we use variable *I^m^* to track the *m*-th observed feature. *I^m^* is the cumulation of $r_{t}^{m}$ and changes over time. When the observed landmark of the preprocessing set is outside the detection range of the sensor, $r_{t}^{m}$ is set to −1; When the observed landmark is in the detection range of the sensor and the landmark is re-observed, $r_{t}^{m}$ is set to 1; When the observed landmark is in the detection range of the sensor but the landmark is not re-observed $r_{t}^{m}$ is set to 0:
  (8) $$I^{m}=\sum_{n}r_{n}^{m}(r_{n}^{m}{|\text{n}}_{\text{k}},\theta_{\text{n}})$$
  If *I_m_* ≥ 2, the landmark will be added to the map and if *I_m_* ≤ 0, the landmark will be discarded. That is, acceptance gate is 2 and discard gate is 0. That is to say, if the landmark is detected twice, it is considered to exist in the map; but if the times it is correctly detected are less than the times it is wrongly detected, the landmark is considered to be fake and should be removed from the map.
  Each time only landmarks in the local area around the robot can be re-observed and thus we only need to make the observations associated with the landmarks in the local map. The local map can be determined according to the detection range of the sensor and the robot pose using Equation (9) where (*m_x_*,*m_y_*) are the coordinates of a landmark and (*x*,*y*,*φ*) is the robot pose, *R* is the used detection range of the sensor, *υ* and Δ are the range and heading angle compensation values, respectively. Equation (9) ensures that the landmark is in the scanning radius and sector of the sonar. For example, the scanning distance of the sonar can be set as 100 m (the maximum range of the Tritech sonar is 300 m), and the scanning sector is 180°, then R = 100 and (*m_x_* − *x*) cos*φ* + (*m_y_* − *y*) sin*φ* > −*R*. The sonar sensor has a systematic deviation, so we expand the local map by a compensation value to prevent omitting landmarks:
  (9) $$\begin{array}{c} \sqrt{{\left(m_{x}-x\right)}^{2}+{\left(m_{y}-y\right)}^{2}}<R+\upsilon \\ (m_{x}-x)\cos\varphi +(m_{y}-y)\text{sin}\varphi >-\Delta \cdot R \end{array}$$
- Calculating Importance Weights of Each Particle
  Before importance resampling, the importance weights must be calculated. The proposal distribution *p*(**s**^t−1^‖**u**^t−1^,**n**^t−1^,**z**^t−1^)*p*(*s_t_*‖**s**^t−1^,**u**^t^,**z**^t^,**n**^t^) do not match the desired posterior *p*(**s**^t^‖**u**^t^,**z**^t^,**n**^t^). The importance weight of each particle is calculated as follows:
  (10) $$w_{t}^{n}=\frac{\begin{array}{cc} \text{target} & \text{distribution} \end{array}}{\begin{array}{cc} \text{proposal} & \text{distribution} \end{array}}=\frac{p(s^{t,n}|z^{t},u^{t},n^{t})}{p(s^{t-1,n}|u^{t-1},n^{t-1},z^{t-1})p(s_{t}^{n}|s^{t-1},u^{t},z^{t},n^{t})}$$
- Rank-Based Resampling
  Resampling, which tends to eliminate particles with low weights, plays an important role but causes the particle depletion problem in FastSLAM. The literature [30,31] has proposed the RBR algorithm which is a resampling algorithm from the rank-based selection in genetic algorithms (GA). This algorithm is proposed to maintain particle diversity as long as possible. It determines the rank of each particle in accordance with its weight and then assigns it a selection probability. It gives the maximal selection probability to the particle with highest weight due to its first rank, and the minimal selection probability to the one with lowest weight due to its last rank. When the number of particles is fixed, the minimal selection probability and the maximal should satisfy the condition that their sum is less than 2, and the selection probability usually has a value ranging between 1 and 2. With the selection probabilities, the RBR performs one of the stratified resampling algorithms. The RBR neglects the relative information among the weights and assigns selection probabilities on the base of their ranks.

In our algorithm, a rank-based resampling algorithm is adopted from the rank-based selection in GA to resolve the particle depletion problem. Differing from RBR algorithm, our algorithm generates new particles with two selected particles, rather than simply by replacement using a selected particle, and that way will ensure better particle diversity. The pseudo code of our resampling algorithm is shown in Table 1. The FastSLAM algorithm considers the “effective sample size” to decide whether to do resampling, this will result in the fact that FastSLAM executes resampling too many times so that the diversity of the particle distribution is reduced and the consistency gets worse [22]. Modified-FastSLAM considers the number of effective particles, the particle weights' covariance, and every particle's residual consistency [32] to check whether it is the right time to do resampling. All the algorithms are executed on the Matlab R2009a platform.

#### 3.2. Simulation Experiments

In this section, simulation experiments are carried out to evaluate the performance of the modified-FastSLAM algorithm in comparison with the original method. The FastSLAM2.0 simulator obtained from [36] is utilized in this secption, and it is taken as original FastSLAM or benchmark algorithm. The state of the robot can be modeled as (*x*,*y*,*φ*) where (*x*,*y*) are the Cartesian coordinates and *φ* is the orientation to the global environment, respectively. The kinematics equations for the mobile robot are in the following form:

(11) $$\left[\begin{array}{l} x_{t}\\ y_{t}\\ \varphi_{t} \end{array}\right]=\left[\begin{array}{l} x_{t-1}+v_{t}\times dt\times \cos(\varphi_{t-1}+G)\\ y_{t-1}+v_{t}\times dt\times \sin(\varphi_{t-1}+G)\\ \varphi_{t-1}+v_{t}\times dt\times \sin(G)/B \end{array}\right]+W_{t}$$

where **u** = [*v* G] is the control input consist of a velocity input and a steer input and B is the wheel-base of the vehicle. *W_t_* is the process noise and assumed to be Gaussian. The observation sensor can provide a measurement of rang *r* and bearing *Ψ* to the observed feature. The observation model is:

(12) $$\left[\begin{array}{c} r\\ \psi \end{array}\right]=\left[\begin{array}{c} \sqrt{{\left(m_{x}-x_{t}\right)}^{2}+{\left(m_{y}-y_{t}\right)}^{2}}\\ \tan^{-1}(\frac{m_{y}-y_{t}}{m_{x}-x_{t}})-\varphi_{t} \end{array}\right]+N_{\text{t}}$$

where (*m_x_*,*m_y_*) are the landmark coordinates and *N_t_* is the observation noise. The environment explored by the robot is a region of 200 m × 200 m size, and the robot initially begins at point (0,0). The relevant parameters in the simulation are listed at Table 2.

**Figure 4.**
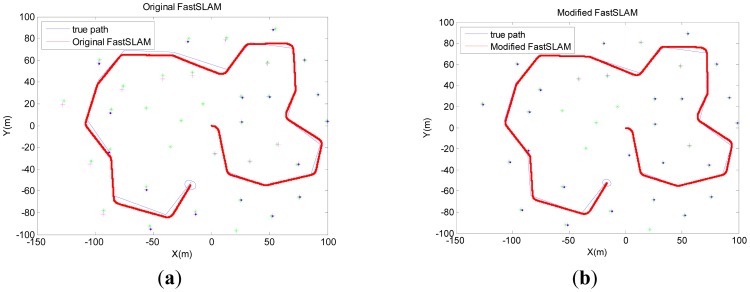
Results of FastSLAM and modified-FastSLAM obtained by averaging over 50 Monte-Carlo trials.

The algorithms are run with 50 particles while the number of effective particles is three-quarters of the total number of particles. Results in the following are obtained by averaging over 50 Monte-Carlo trials. Pose errors, RMS, NEES, will be produced to evaluate the estimation performance in the next section. Figure 4 presents the simulation results of the original FastSLAM and the modified-FastSLAM. The modified-FastSLAM can maintain a better estimation of path and landmarks.

**Figure 5.**
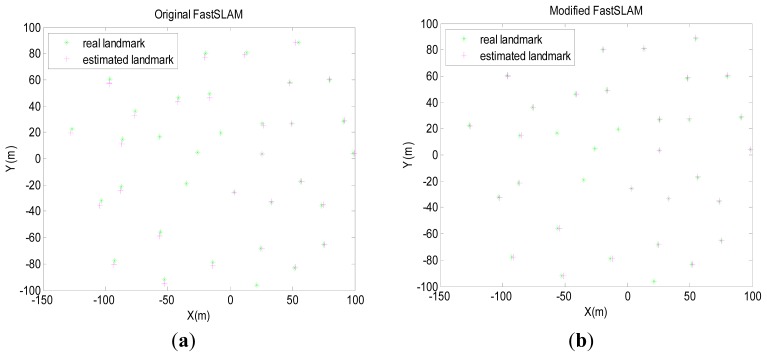
Data association results.

The data association performances of the two algorithms are shown in Figure 5. It can be seen that the modified algorithm incorporating negative information into FastSLAM results in a measurable increase in the accuracy of the resulting map.

**Figure 6.**
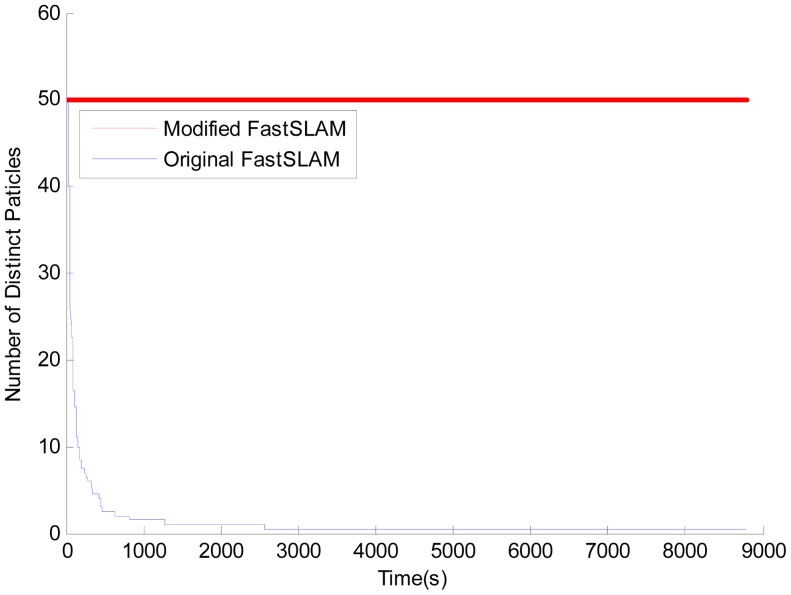
Number of distinct particles over time. The particle number used is 50.

Comparison of the loss of particle diversity between original FastSLAM and modified FastSLAM is shown in Figure 6. The number of distinct particles is counted after every resampling process. As the figure shows, the original version shows faster convergence, but for the modified one using our new resampling algorithm the number of distinct particles is quite steady. With the large variance of particles' weights, the rank-based resampling re-assigns the selection probability based on the rank of each particle, and consequently attains better particle diversity and resolves the particle depletion problem.

**Figure 7.**
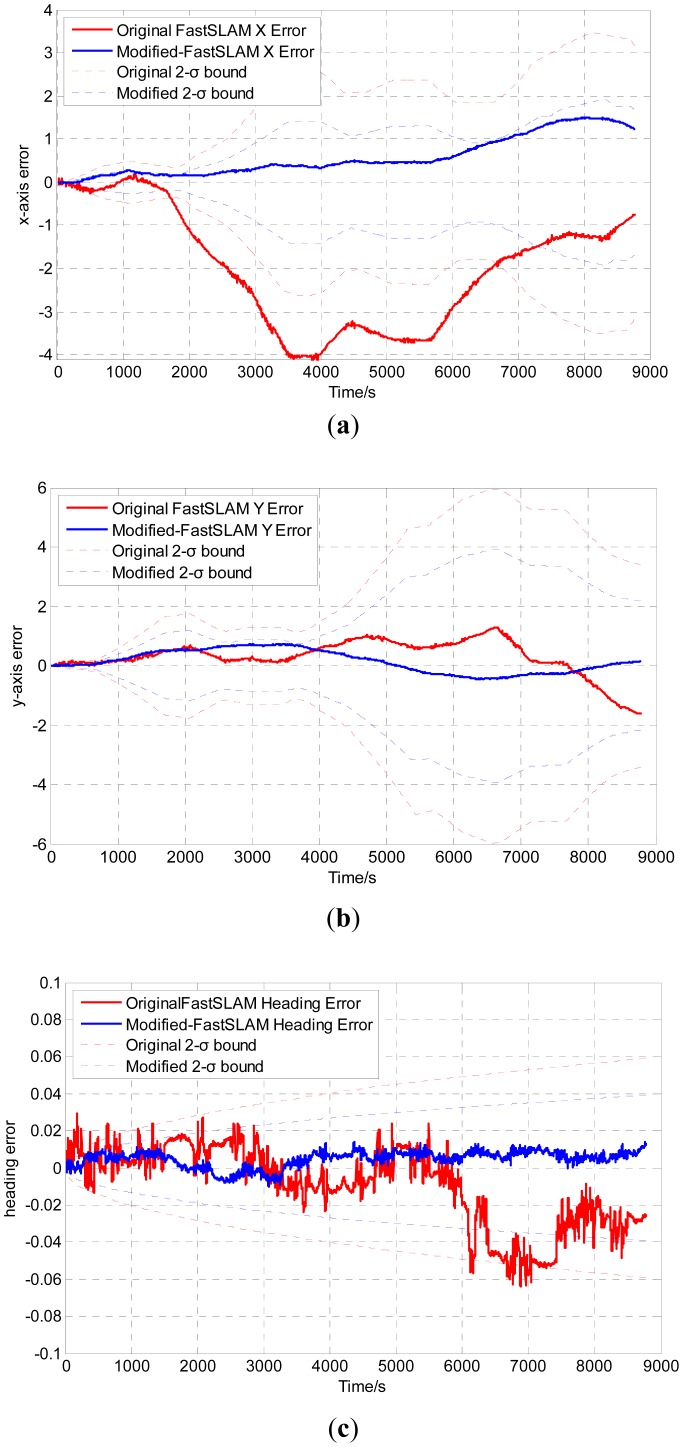
Estimation pose error with 2-σ uncertainty bound of the original FastSLAM and the modified FastSLAM algorithm.

The robot pose errors are shown in Figure 7, where the solid lines denote errors between the estimation results and the ground truth, and the dotted lines stand for the 2-σ uncertainty bounds. Compared with the 2-σ bounds, the estimation error of the modified algorithms is always within the 2-σ bounds, while the original algorithm will diverge beyond the uncertainty range. The results show that the modified algorithm can improve the estimation accuracy and outperforms the original FastSLAM algorithm.

**Figure 8.**
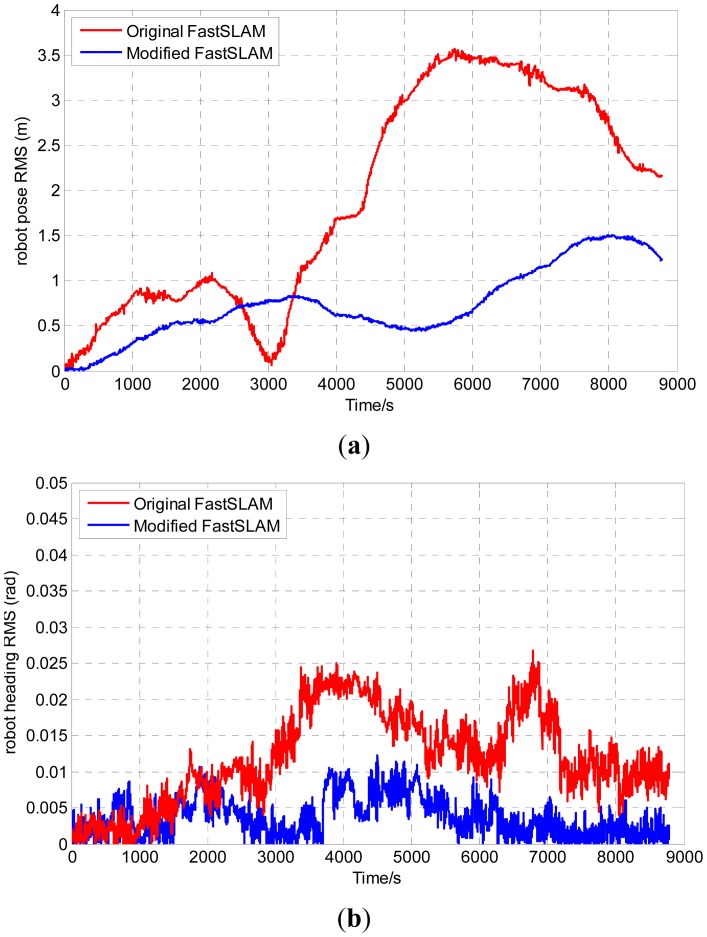
RMS error of the two algorithms.

**Figure 9.**
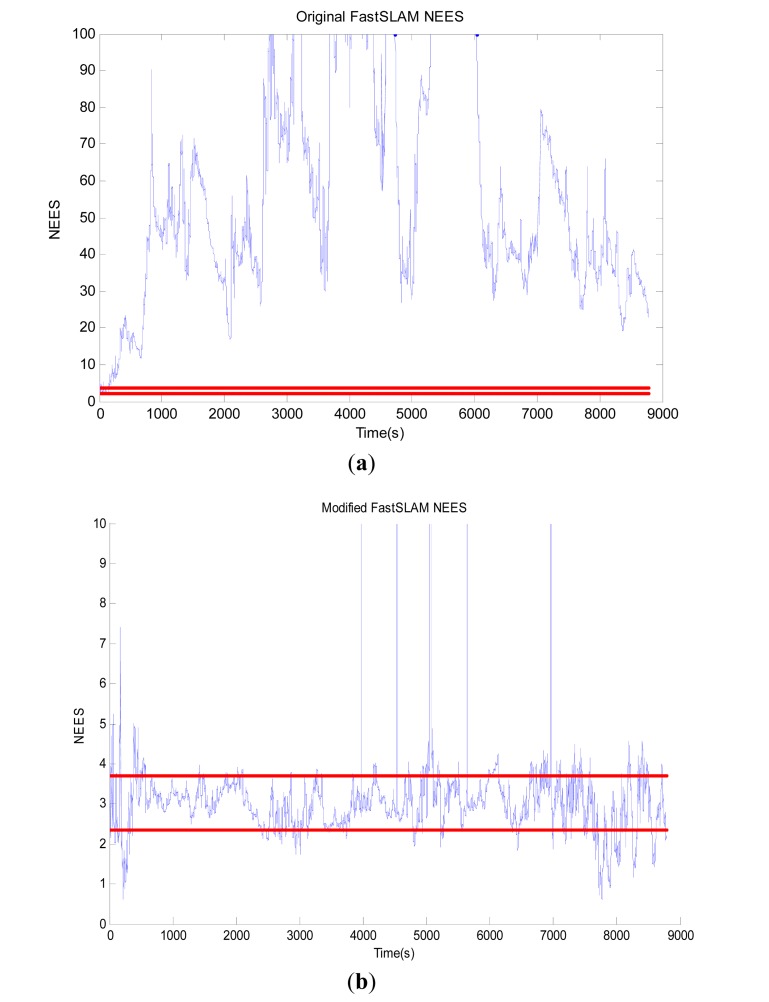
Average NEES of the two algorithms.

In order to evaluate the consistency of the two algorithms, we adopt the *root mean square* (RMS) and the *normalized estimation error squared* (NEES) to test whether the modified-FastSLAM is consistent or not over the long term and compare its results with those of the original one. The comparison results are shown in Figures 8 and 9.

RMS errors of robot position and heading are shown in Figure 8. As the figure shows, the RMS errors of the original version and the modified one are in the interval (0, 4) and (0, 1.6), respectively. Obviously, the RMS error of the modified-FastSLAM is more satisfactory than the original one.

We use the average NEES to characterize the filter performance. NEES at time *t* is obtained as:

(13) $$\xi t={\left(xt-\hat{x}t\right)}^{T}P_{t}^{-1}\left(xt-\hat{x}t\right)$$

where *x*ˆ*_t_* and *P_t_* are the estimated mean and covariance of particles at time *t*. A measure of filter consistency is analyzed by examination of the average NEES over *N* Monte Carlo trials of the filter. Under the assumptions that the filter is consistent and approximately linear-Gaussian, *ξ_t_* is *χ*^2^(chi-square) distributed with the dim (*x_t_*) degrees of freedom. Given *N* Monte-Carlo trials, the average NEES is:

(14) $${\bar{\xi }}_{t}=\frac{1}{N}\sum_{i=1}^{N}\xi_{t}^{i}$$

The dimension of the robot pose is 3, with N = 50, the 95% probability concentration region for *ξ̄_t_* is bounded by the interval [2.36, 3.72]. The average NEES of the two FastSLAM algorithms is shown in Figure 9, where the blue line depicts the NEES value at each time, and the two horizontal red lines represent the low and high bounds for the average Chi-Square distribution. Obviously, the original FastSLAM is not consistent as is shown in Figure 9(a). To be more precise, after about 3000 time steps, the filter becomes optimistic. While the consistency of the estimated vehicle pose keep much better in the modified FastSLAM algorithm as Figure 9(b) shows. Consequently, the modified algorithm can remarkably improve the consistency and has a good robustness.

All of the experimental results confirm the effectiveness of the modified algorithm. Its main advantage is that the proposed method is more consistent than the original FastSLAM. Also, the deviation of the modified FastSLAM algorithm is far less than that of the original method. In addition, the modified-FastSLAM has good robustness. On the whole, the modified-FastSLAM outperforms the original one.

### 4. Sea Trial

#### 4.1. SLAM for C-Ranger

In the *C-Ranger* AUV system, the posterior can be described as:

(15) $$p(s^{t},v_{x},v_{y},\Theta ,|z^{t},u^{t},n^{t}$$

where **u** = [*w*,*a_x_*,*a_y_*]*^T^* is the *C-Ranger's* control input which contains the accelerations *a_x_* and *a_y_* respectively, and the angular velocity of the vehicle *w*.

State vector **X** = [**s** *v_x_ v_y_*]*^T^* consists of vehicle pose **s** = [*x*,*y*,*φ*]*^T^* and velocities of the vehicle *v_x_*, *v_y_*. In order to obtain better estimates and reduce the computational cost, the state equation can be divided into the nonlinear portion **X**^n^ = [*x y*]*^T^* + **W**^n^, and the nonlinear portion **X**^1^ = [*φ v_x_ v_y_*]*^T^* + **W**^1^, where **W**^n^ = [*N_x_*,*N_y_*]*^T^* is the additive zero mean uncorrelated Gaussian motion disturbances with covariance *Q^n^*, and **W^l^** =[*N_φ_*,*N_x_*,*N_y_*]*^T^* is the additive zero mean uncorrelated Gaussian motion disturbances with covariance *Q^l^*. The superscripts *l* and *n* stand for “linear” and “nonlinear”, respectively. The AUV's motion model is shown as follows:

(16) $$\begin{array}{l} X_{t}={\left[X_{t}^{n}X_{t}^{l}\right]}^{T}\\ X_{t}^{n}\Rightarrow \{\begin{array}{l} x\begin{array}{c} t \end{array}=x\begin{array}{c} t-1 \end{array}+(vx\begin{array}{c} , \end{array}t-1\times \Delta t+0.5\times ax,\begin{array}{c} t-1 \end{array}\times \Delta t\times \Delta t)\times \cos(\varphi_{t-1})-(vy\begin{array}{c} , \end{array}t-1\times \Delta t+0.5\times ay,\begin{array}{c} t-1 \end{array}\times \Delta t\times \Delta t)\times \sin(\varphi_{t-1})+Nx,t-1\\ y\begin{array}{c} t \end{array}=y\begin{array}{c} t-1 \end{array}+(vx\begin{array}{c} , \end{array}t-1\times \Delta t+0.5\times ax,\begin{array}{c} t-1 \end{array}\times \Delta t\times \Delta t)\times \sin(\varphi_{t-1})+(vy\begin{array}{c} , \end{array}t-1\times \Delta t+0.5\times ay,\begin{array}{c} t-1 \end{array}\times \Delta t\times \Delta t)\times \cos(\varphi_{t-1})+Ny,t-1 \end{array}\\ X_{t}^{n}\Rightarrow \{\begin{array}{l} \varphi_{t}=\varphi_{t-1}+w\begin{array}{c} t-1 \end{array}\times \Delta t+N_{\varphi ,t-1}\\ vx,\begin{array}{c} t \end{array}=vx,\begin{array}{c} t-1 \end{array}+ax,\begin{array}{c} t-1 \end{array}\times \Delta t+Nvx,t-1\\ vy,\begin{array}{c} t \end{array}=vy,\begin{array}{c} t-1 \end{array}+ay,\begin{array}{c} t-1 \end{array}\times \Delta t+N_{v_{y}},t-1 \end{array} \end{array}$$

Like the state vector, the observation vector also is divided into two parts **Z** = [**Z**^n^ **Z**^1^]*^T^*. **Z**^n^ is from sonar and **Z**^1^ is from compass and DVL. To map its environment, the AUV senses landmarks by imaging sonar. It is able to measure range and bearing to landmarks denoted as **Z**^n^ = [*r*,*φ*]*^T^*. The observed landmark position in the global coordinate frame is denoted as [*m_x_*,*m_y_*]*^T^*, and the observation model of the sonar part is:

(17) $$Z_{t}^{n}=g(s_{t},m)=\left[\begin{array}{c} r(s_{t},m)\\ \psi (s_{t},m_{t}) \end{array}\right]=\left[\begin{array}{c} \sqrt{{\left(m_{x}-x_{t}\right)}^{2}+{\left(m_{y}-y_{t}\right)}^{2}}\\ tan^{-1}(\frac{m_{y}-y_{t}}{m_{x}-x_{t}})-\varphi_{t} \end{array}\right]+N_{t}^{n}$$

where $N_{t}^{n}$ is additive zero mean uncorrelated observation errors vector with covariance *R^n^*.

The compass and DVL measurements $Z_{t}^{l}$ are:

(18) $$Z_{t}^{l}=\left[\begin{array}{l} \varphi_{t}\\ v_{x,t}\\ v_{y,t} \end{array}\right]+N_{t}^{l}$$

where *φ* is the angle from the digital compass, *v_x_*, *v_y_* are the velocities of the vehicle provide by DVL, and $N_{t}^{l}$ is the measurement noise with zero mean Gaussian distributed noise vector with covariance *R^l^*.

**Z**^n^ is independent of **X**^1^, and **Z**^1^ is independent of **X**^n^, using Bayes' theorem the posterior can be factored into Equation (19) shown as follows:

(19) $$p(X_{t}^{l}|X^{n,t},Z^{l,t},u^{t},n^{t})p(X^{n,t}|Z^{n,t},u^{t},n^{t})\prod_{i=1}^{N}p(\theta_{i}|X^{n,t},Z^{n,t},u^{t},n^{t})$$

For the whole system, the first term in Equation (19) is linear-Gaussian [33], so optimal results can be obtained using Kalman filter, and the last two terms will be handled using a particle filter. The whole algorithm can be summarized as is Table 3.

Firstly, initialize state vector. Employ AHRS and gyro data to predict the AUV's next location. Then extract point features from sonar data to update or augment the map. After that, the robot pose will be updated and importance weight will be computed. Lastly do resampling. It is a recursive process. The linear state **X**^1^ is estimated using a standard Kalman filter. It is updated twice. First it is updated by using observation **Z**^1^, and then **X**^n^ is treated as an observation to update **X**^1^.

#### 4.2. Sea Trial

The experiment was performed at Tuandao Bay (Qingdao, China) and the sea trial dataset samples were obtained from the on-board sensors introduced in Section 2. A sonar operating with 180° scanning-sector and 100 m-range is used to sense the environment, and features in the environment are extracted and used to build the global map of the environment based on the algorithm proposed in this paper.

**Figure 10.**
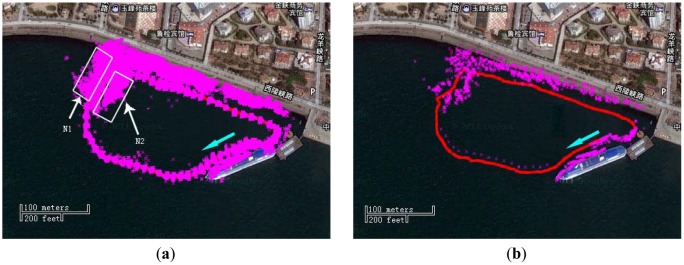
(**a**)The satellite image combined with the sonar image without sparsification (the pink points) and the GPS path (the red line); (**b**) The figure after sonar feature extraction and sparsification.

In Figure 10, a starting point with direction is marked with a cyan arrow. There are some underwater drainage pipes at the location N1 and several little boats at place N2. From the Figure 10(a), it can be seen that the sonar image is well matched with the satellite image. But there are also some points in or nearby the trajectory, probably generated because of the beams reflected by the sonar head shell and some moving objects. The features are very dense, if they are used for data association, the efficiency will be very low. In order to improve the efficiency of SLAM, a process of sonar feature extraction and sparsification had been conducted [26]. The resulting figure is shown in Figure 10(b). From this figure, it can be noted that the number of features is reduced significantly, and there are few noise points in or near the trajectory.

**Figure 11.**
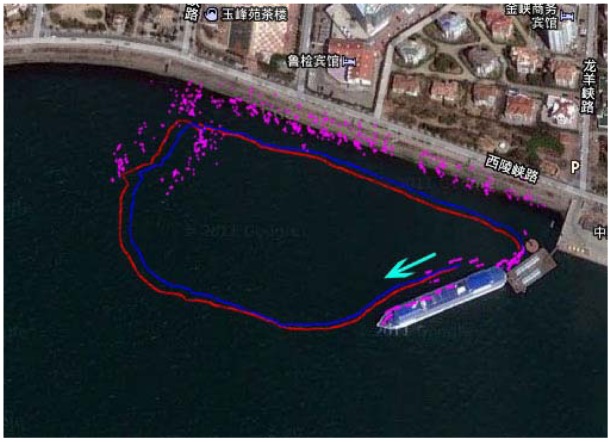
The modified FastSLAM resulting map combined with the environmental map.

The resulting map of the sea trials combined with the environmental map is shown in Figure 11, where the magenta points indicate the point features, the red line represents the GPS trajectory which acts as the ground truth to compare the effectiveness of algorithms, and the blue solid line represents the path from the modified FastSLAM. It can be noted that the number of point features decreased because of data association, and the point features match well with the landmarks in the real environment. In addition, since any features would be deleted if observed only once in the data association process, most of noise points in or near-by the GPS trajectory are removed.

**Figure 12.**
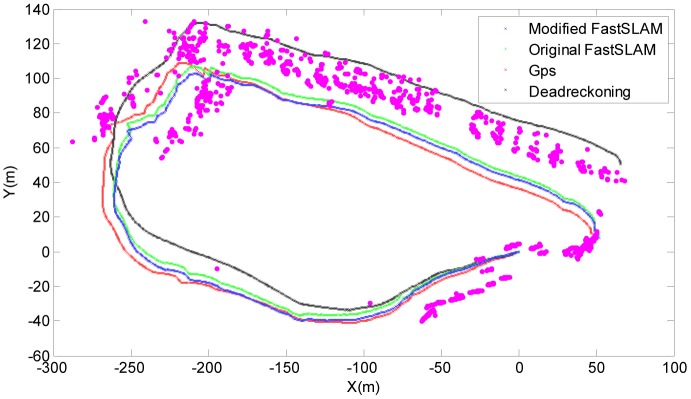
Comparison of the trajectories for GPS, Dead-Reckoning, Original FastSLAM and Modified-SLAM by averaging over 50 independent runs.

Figure 12 is a comparison of the trajectories for GPS, dead-reckoning, FastSLAM and the modified-FastSLAM algorithms obtained by averaging over 50 independent runs. Obviously, the deviation of original FastSLAM (the green line) relative to GPS is smaller than that of dead-reckoning (the black line) and greater than that of modified one (the blue line).

**Figure 13.**
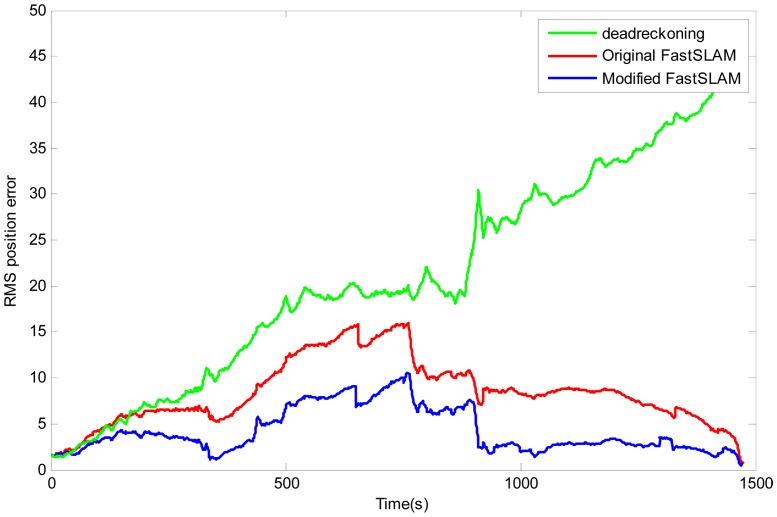
The position RMSE of Dead-Reckoning, original FastSLAM and modified-FastSLAM by averaging over 50 independent runs.

In order to get a clear demonstration of the presented algorithm, the position RMS errors by the dead-reckoning, the original FastSLAM (the red line) and the modified-FastSLAM (the blue line) are compared in Figure 13. It can be noted clearly that the overall trend of the error by dead-reckoning is divergent. The curves of the original FastSLAM and modified-FastSLAM have similar trends, and they both increase in the beginning phase due to the cumulative errors, and then they will decrease because landmarks are repeatedly observed so that the trajectory and the map are updated. It is obvious that the position error of the Modified-FastSLAM is less than that of the original FastSLAM, the maximum error of the original FastSLAM trajectory is up to about 16 m, while the Modified-FastSLAM is only about 10 m.

**Figure 14.**
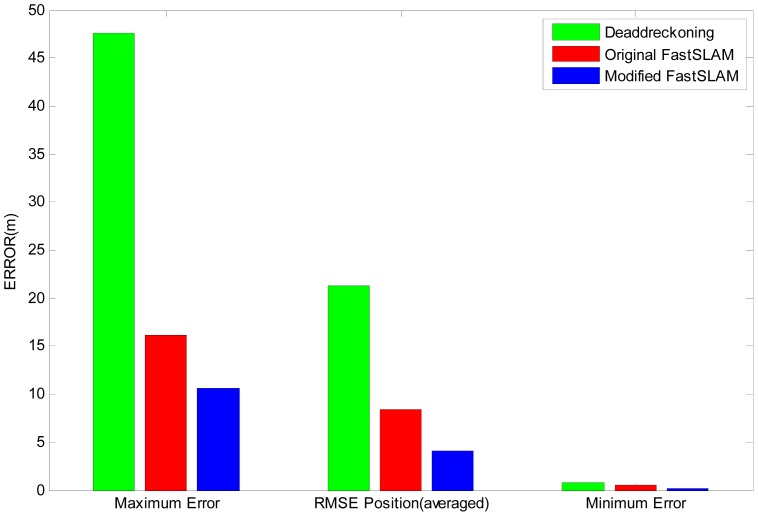
. RMSE summarization of Dead-Reckoning, original FastSLAM and modified-FastSLAM over 50 runs.

To further evaluate the performance of the Modified-FastSLAM, the RMS errors of AUV positions over the results of 50 runs are presented in Figure 14.

The maximum errors, the minimum errors and the averaged RMSE of the algorithms are compared in this figure. It can be seen clearly that the errors of modified-FastSLAM are much smaller than those of the other two algorithms. The standard deviations of estimation errors are summarized in Table 4.

**Figure 15.**
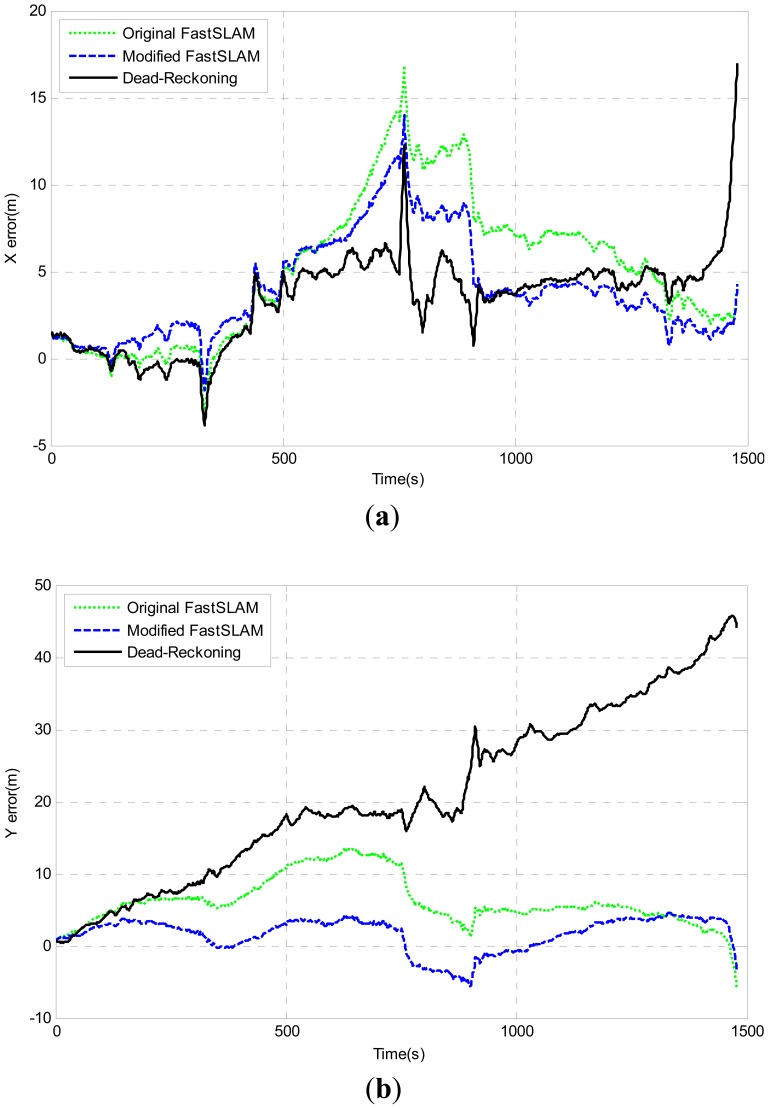
Pose errors between estimated pose and GPS ground truth. (**a**) Easting x-axis; error; (**b**) Northing (Y-axis) error; (**c**) Heading-angle error.

The Modified-FastSLAM also shows the best performance compared with the other two algorithms. Furthermore, the comparison of pose errors on x-axis, y-axis and heading angle are shown in Figure 15. The above results can basically validate that the better performance of the modified-FastSLAM algorithm.

### 5. Discussion

Although the Modified-FastSLAM algorithm is better than the original FastSLAM algorithm according to simulations and field experiments, there are still some problems. Compressing point features in the feature extraction process is time-consuming as a result of the large number of point features. It is also a time-consuming process to determine resampling when compared with standard FastSLAM. However, information compression of point features can reduce the time-cost of data association eventually; especially the employed resampling condition can limit the frequency of resampling, so the total computational cost of the algorithm does not increase.

The proposed algorithm does not fundamentally solve the consistency problem though it is more consistent than the original one, so it might face the problem in applications involving large scale environments. Some algorithms have been proposed to deal with this problem. Among them, the submap-based SLAM is a more effective one. In future works we will try to apply the modified-FastSLAM to combine with EIFSLAM. The modified-FastSLAM can be used to produce local maps, which are periodically fused into an EIFSLAM algorithm. Modified-FastSLAM may avoid linearization of the robot model during operation and provide a robust data association, while EIFSLAM would improve the overall computational speed.

The mechanical-scanning sonar used on the *C-Ranger* is two-dimensional type. It can only determine the location of the targets in the two-dimensional plane, but can not determine the height of the target. The targets in the acoustic image can not be clearly identified what they are, and that is not helpful for the vehicle to avoid obstacles, so a 3D sonar will be installed on the AUV platform in future for a better description of the undersea environment.

### 6. Conclusion

This paper describes the *C-Ranger* experimental platform and introduces the implementation of modified-FastSLAM navigation. The modified-FastSLAM brings together the advantages of different methods that have been proposed to optimize the standard FastSLAM. It employs the data association method which combines the single particle maximum likelihood method with the modified negative evidence method, uses the rank-based resampling to overcome the particle depletion problem. The experimental results performed in the simulation demonstrate the robustness and the accuracy of the proposed algorithm, and the sea trial results show the modified-FastSLAM employed for the navigation of *C-Ranger* AUV is actually more effective and accurate compared with the traditional methods.

## Figures and Tables

**Table 1.** Resampling algorithm.

If Neff<Nparticles*0.75&&Weight_covariance>Mean_weight && residual_inconsistency
newparticles=resampling(Particles,weight,Nparticles,Nnew_particles);
end
Function newparticles=resampling(Particles,weight,Nparticles,Nnew_particles)
Nr=zeros(1,Nparticles);
U=rand()/Nparticles;
Cmax=1+rand();
Cmin=2-Cmax;
Ws,rank=sort(w,'descend');
for i=1 to Nparticles
K=rank(i);
ps(k)=(Cmax-(Cmax-Cmin)*(i-1)/(Nparticles-1))/Nparticles;
Nreplace(i)=fix((ps(i)-U)*N)+1;
U=U+Nreplace(i)/N-ps(i);
end
index=find(Nr>0);
Len=length(jj);
for i=1:Nnew_particles
index1=index(round(rand()*Len));
index2=index(round(rand()*Len))å
Rand_value=rand();
New_particles(i)=Rand_value*particles(index1)+(1- Rand_value)*(particles(index2);
end

**Table 2.** Parameter settings used in the simulation.

**wheelbase of robot**	4 m	**observation noise**	(0.1 m/s, 0.1 deg)
**speed of robot**	3 m/s	**control frequency**	40 HZ
**maximum steering angle**	30 deg	**observation frequency**	5 HZ
**control noise**	(0.3 m/s, 3 deg)	**maximum of range-bearing sensor**	30 m

**Table 3.** The SLAM algorithm in the C-Ranger AUV.

**Function FastSLAM_SIM()**
for i=1:num_of_particles
Initialization particles
end for
while sensors data are ready do
for t=1 to num_of_particles
Predict the state **X**
Compute the weight of each particle and normalize
Update **X**^l^ using **X**^n^
end for
/*Extract point features from sonar data for mapping and update the vehicle's state.*/
if sonar data are ready then
for i=1 to num_of_particles
Particles(i),n=data_association
/*n is the data association results.*/
for num =1:length(n) do
if n(num) is a new feature then
Augment the map Θ
else
Update **X**^n^ using particle filter
end if
end for
If ***I****_m_*<0
remove m-th spurious feature
end if
Update **X**^l^ using **X**^n^
end for
end if
Employ Rank-based resampling to do resampling
end while

**Table 4.** Standard Deviation of Estimation Errors.

**Type**	**Std. Deviation (m)**

Deadreckoning	0.1979
Original FastSLAM	0.0846
Modified FastSLAM	0.0595
